# The Prohormone Proinsulin as a Neuroprotective Factor: Past History and Future Prospects

**DOI:** 10.3389/fnmol.2018.00426

**Published:** 2018-11-26

**Authors:** Flora de Pablo, Catalina Hernández-Sánchez, Enrique J. de la Rosa

**Affiliations:** ^1^3D Lab, Development, Differentiation and Degeneration, Centro de Investigaciones Biológicas, Consejo Superior de Investigaciones Científicas (CIB/CSIC), Madrid, Spain; ^2^Centro de Investigación Biomédica en Red de Diabetes y Enfermedades Metabólicas Asociadas (CIBERDEM), ISCIII, Madrid, Spain

**Keywords:** proinsulin, insulin receptor isoforms, IR-A, neuroretina, photoreceptors, retinitis pigmentosa, neurodegeneration, neuroprotection

## Abstract

Proinsulin was first identified as the primary translation product of the insulin gene in Donald Steiner’s laboratory in 1967, and was the first prohormone to be isolated and sequenced. While its role as an insulin precursor has been extensively studied in the field of endocrinology, the bioactivity of the proinsulin molecule itself has received much less attention. Insulin binds to isoforms A and B of the insulin receptor (IR) with high affinity. Proinsulin, in contrast, binds with high affinity only to IR-A, which is present in the nervous system, among other tissues and elicits antiapoptotic and neuroprotective effects in the developing and postnatal nervous system. Proinsulin specifically exerts neuroprotection in the degenerating retina in mouse and rat models of retinitis pigmentosa (RP), delaying photoreceptor and vision loss after local administration in the eye or systemic (intramuscular) administration of an adeno-associated viral (AAV) vector that induces constitutive proinsulin release. AAV-mediated proinsulin expression also decreases the expression of neuroinflammation markers in the hippocampus and sustains cognitive performance in a mouse model of precocious brain senescence. We have therefore proposed that proinsulin should be considered a functionally distinct member of the insulin superfamily. Here, we briefly review the legacy of Steiner’s research, the neural expression of proinsulin, and the tissue expression patterns and functional characteristics of IR-A. We discuss the neuroprotective activity of proinsulin and its potential as a therapeutic tool in neurodegenerative conditions of the central nervous system, particularly in retinal dystrophies.

## The Discovery of Proinsulin

A seminal 1967 article (Steiner et al., 1967) describing proinsulin biosynthesis introduced this molecule as a paradigm of precursor protein production and processing. The authors demonstrated that the mature insulin molecule consists of two polypeptidic chains (designated A and B) joined by two disulfide bonds, plus an intrachain disulfide bond in chain A. These two chains, and the cleaved C-peptide, are generated in the pancreas by specific proteolytic processing of the single-chain precursor molecule, proinsulin, by cellular proprotein convertases. Low levels of unprocessed proinsulin were subsequently described in circulating plasma (Melani et al., 1970). The discovery and characterization of the biochemical features of proinsulin brought about a marked change in the general understanding of hormone and neuropeptide biosynthesis (reviewed in Steiner, 2011). This foundational research on proinsulin and convertases (Rouillé et al., 1995) has had a long-lasting impact on protein crystallography and the understanding of the relationship between protein sequence, structure, folding and function. The impact of Steiner’s proinsulin findings, as well as his pioneering studies on the evolutionary biology of insulin-like proteins, are summarized in an article (Weiss and Chan, 2015) commemorating his contributions to science published shortly after his death in 2014.

The discovery of proinsulin represents an important milestone in peptidic hormone biology (Rholam and Fahy, 2009). Steiner’s descriptions of proinsulin synthesis and processing and the regulation of insulin secretion by pancreatic beta cells form the basis for our current understanding of prohormone storage in secretory granules and secretagogue-induced release (Steiner et al., 2009). Indeed, similar processes have since been described in other glands. Shortly after the discovery of proinsulin, Moore and colleagues showed that introduction of proinsulin cDNA into a cell lacking secretory machinery (e.g., a fibroblast) led to the secretion of proinsulin only; the fibroblasts did not store the protein and the secretion rate was constitutive and unaffected by secretagogues (Moore et al., 1983). Interestingly, in all species tested the plasma half-life of proinsulin was more than twice that of insulin, and minimal conversion of proinsulin to insulin or other intermediate forms occurred outside the pancreas (Robbins et al., 1984). Subsequent studies confirmed that the processing of other protein precursors, including proglucagon, prosomatostatin and proopiomelanocortin, was dependent on cell-specific expression of the corresponding prohormones and their convertases, and on trafficking via the secretory pathway (Douglass et al., 1984; Rholam and Fahy, 2009).

## Prepancreatic and Extrapancreatic Expression of the Insulin Gene

For several decades it remained unclear whether the insulin gene (which we will refer as the “proinsulin gene”) was expressed in extrapancreatic tissues (Eng and Yalow, 1981; Kojima et al., 2006) or before pancreatic differentiation in embryos (de Pablo et al., 1990; de Pablo and de la Rosa, 1995; Morales et al., 1997). Experiments in developing chicks and mice provided evidence of gene expression in nonpancreatic tissues, suggesting that proinsulin could be secreted and act as a signaling factor (Hernández-Sánchez et al., 2006).

Since the 1970s, studies have described the extrapancreatic expression of proinsulin/insulin, particularly in the nervous system. Jesse Roth’s research group conducted pioneering work that demonstrated insulin immunoreactivity in rat brain (Havrankova et al., 1978), a finding supported by a later study showing that insulin binds to the abundant insulin receptors (IRs) in brain (Schwartz et al., 1992). Not until several years later was it shown that brain proinsulin/insulin expression is a consequence of neuronal expression of the proinsulin gene; cells containing proinsulin mRNA were identified in the periventricular area of the rat brain (Young, 1986) and in neurons (Devaskar et al., 1994). Extensive studies in chick embryos revealed developmentally regulated proinsulin mRNA expression during gastrulation and neurulation, in the neuroretina during proliferation and differentiation, and in the embryonic brain, liver and eye (Hernández-Sánchez et al., 1995; Morales et al., 1997; Alarcón et al., 1998). Translation of prepancreatic proinsulin mRNA was found to be tightly controlled in the chick (Hernández-Sánchez et al., 2003), and the low levels of protein produced remained as unprocessed proinsulin (Hernández-Sánchez et al., 1995; Alarcón et al., 1998) and were rapidly secreted. An even more complex situation was uncovered in the developing heart in chick embryos, where expression levels of the translationally active proinsulin transcript (Pro 1B) were much lower than in the neurulating embryo, and an intron-retaining variant with lower translation capacity predominated (Martínez-Campos et al., 2013). Indeed, we found that Pro 1B overexpression, which presumably increases proinsulin signaling in the embryo, led to abnormal cardiac morphogenesis. This finding underscores the importance of strict control of embryonic proinsulin expression and suggests a possible role of proinsulin as a key signaling factor. Proinsulin mRNA has also been detected from embryonic day (E) 13.5 to E18.5 in the mouse retina, and exogenous proinsulin shown to be as effective as insulin or IGF-I in decreasing apoptotic cell death in cultured retinas (Valenciano et al., 2006).

Insulin-like peptides emerged early in evolution. Chan and Steiner (2000) conducted a thorough analysis of the phylogenetic origin of the proinsulin gene and reported its presence in insects, mollusks and nematodes, among other multicellular species. Given their absence in the yeast genome, members of the family of insulin-like factor genes appear to have co-evolved with the appearance of the metazoan branch (Chervitz et al., 1998). As discussed in our review of proinsulin and insulin-like peptides in neural development (de la Rosa and de Pablo, 2011), a remarkable eight insulin-like genes are found in *D. melanogaster* (Nässel and Vanden Broeck, 2016), with cell and stage-specific patterns of expression, and 40 such genes in *C. elegans* (Li et al., 2003; Cornils et al., 2011).

## IR-A, a Proinsulin Receptor: Expression and Signaling in the Nervous System

The IR is a membrane-bound receptor found in all vertebrate cells, and has an α_2_-β_2_ tetrameric structure and intrinsic tyrosine kinase activity. Key mediators of the intracellular effects of insulin include the adaptor proteins IR substrates (IRSs), the lipid kinase PI3K, and the serine/threonine kinase AKT. For a comprehensive overview of the signaling properties of the IR, see Haeusler et al. (2018).

**Figure 1:**
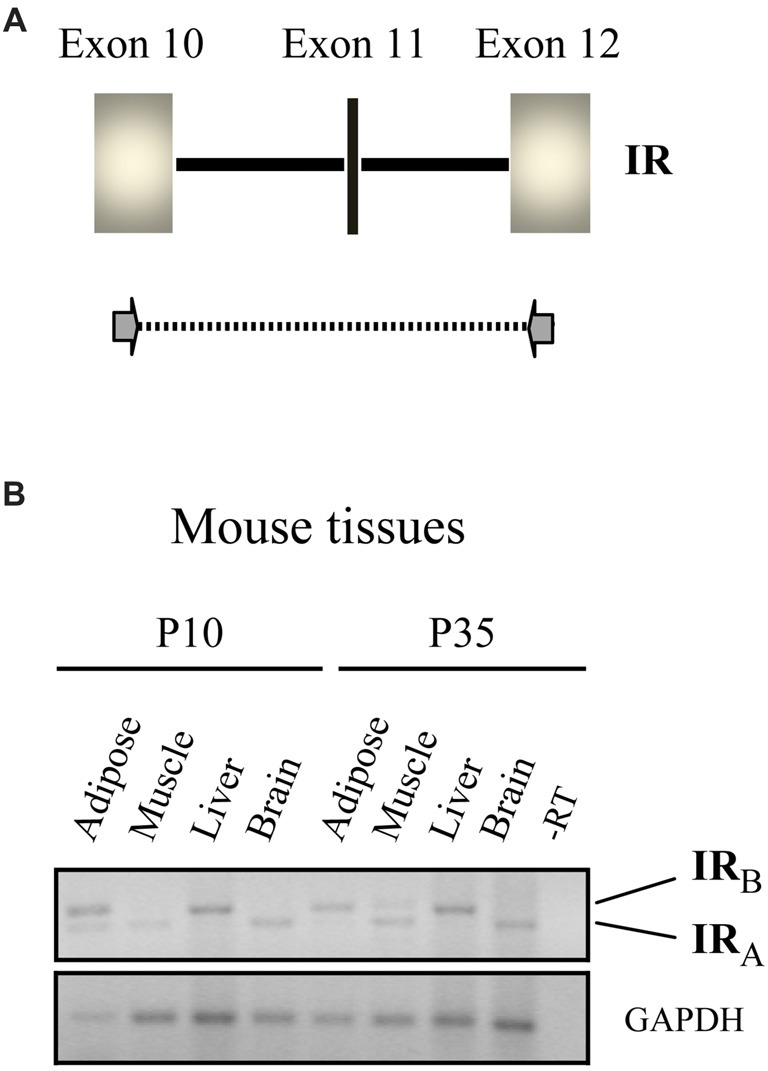
Isoforms of the insulin receptor (IR) expressed in different tissues. **(A)** Schematic representation of the mammalian IR gene, exons 10–12. Exon 11 (in black vertical line) is spliced out during RNA processing in certain tissues. Primers used in the PCR shown in **(B)** are indicated with arrows. **(B)** IR-specific RT-PCR of RNA from the indicated mouse tissues at postnatal day 10 (P10) and P35. The positions of the amplified fragments corresponding to isoforms A and B of the IR are indicated. Note that only the IR-A isoform is expressed in the brain at both time points, whereas the IR-B isoform is expressed in the liver (figure adapted from Hernández-Sánchez et al., 2008).

Alternative splicing of IR gene gives rise to two isoforms, A and B; exon 11, which encodes 12 amino acids, is only found in IR-B (Figure 1A). These isoforms differ in terms of tissue distribution and signaling properties, their expression is tightly regulated and play distinct roles in controlling cell functions (Vienberg et al., 2011). IR-A is the predominant or exclusive form expressed in mammalian fetal tissues, stem cells and adult brain, whereas it is expressed together with IR-B in variable proportion in other tissues (reviewed extensively in Belfiore et al., 2017). In the liver, IRs (mainly IR-B) are involved in regulating metabolism and show a reduced affinity for proinsulin (reviewed in Belfiore et al., 2009), in good agreement with the low metabolic potency of proinsulin (Galloway et al., 1992). Until recently, it was unclear which receptor type mediated the cell pro-survival effects of proinsulin. Studies of cellular expression of IR isoforms A and B revealed that IR-A mediates the downstream cellular effects of proinsulin. Indeed, in cultured cells nanomolar concentrations of proinsulin resulted in comparable stimulation of IR-A phosphorylation and ERK pathway activation to those of insulin (Malaguarnera et al., 2012; Belfiore et al., 2017). Those authors also reported that proinsulin resulted in lesser activation of AKT and failed to bind to IGF-I receptors or hybrid IR/IGF-IR receptors. That proinsulin does not bind to IGF-I receptors in human tissues is well demonstrated (Burguera et al., 1991), and has important implications for proinsulin-based therapies, decreasing the potential unwanted side effects, as discussed further below. In recent studies, dysregulation of the ratio of IR-A/IR-B in various tissues has been associated with insulin resistance, aging and increased proliferative activity (Belfiore et al., 2017).

Remarkably, IR-A, which binds IGF-II with a similar affinity to insulin, is evolutionarily older than IR-B, which first appeared in the mammalian genome (Hernández-Sánchez et al., 2008). In the liver, IR-B is the predominantly expressed isoform, as stated above, whereas in adipose tissue and muscle intermediate expression levels of isoforms A and B are found (Belfiore et al., 2017). By contrast, IR-A is the only isoform expressed in neurons (reviewed in Gralle, 2017). Our studies have only detected expression of IR-A in mouse brain (Figure 1B) and since the retina is also part of the central nervous system, we suspect that it will be the predominant IR type found in this tissue.

IR has been visualized by immunohistochemistry in rods and cones and other cell layers of the mammalian retina (Rodrigues et al., 1988; Gosbell et al., 2002). However, available immunohistochemical reagents do not distinguish between the two IR isoforms, therefore specific studies at the mRNA level need to be performed. Widespread IR-A mRNA expression is observed from early proliferative stages onward in the developing chick embryo retina, in which splicing variants are absent (as in all non-mammalian species; de la Rosa et al., 1994; Hernández-Sánchez et al., 2008). Rajala and coworkers conducted specific functional studies of mammalian IR in photoreceptors (Rajala et al., 2008, 2009, 2013). In rod-specific IR knock-out mice they demonstrated reduced PI3K and AKT signaling in rod photoreceptors, and consequent impairment of retinal function and photoreceptor loss following exposure to bright light (Rajala et al., 2008). Moreover, they found that cultured retina photoreceptors from newborn IR knock-out mice exhibited abnormal development and differentiation, with disorganization of the actin and tubulin cytoskeleton leading to a wide axonal growth cone phenotype (Rajala et al., 2009), and that loss of PI3K signaling in cones resulted in cone degeneration (Rajala et al., 2013). It should be noted however that these studies did not identify the active IR ligand (or ligands) in mouse retina.

## Neuroprotective Effects of Proinsulin in the Brain and the Retina

Studies demonstrating higher concentrations of proinsulin and insulin in the brain than in plasma, as well as developmental regulation of IR expression from early embryogenesis, led researchers to suspect potential autocrine-paracrine effects of both hormones in the nervous system (reviewed in Hernández-Sánchez et al., 2006 and de la Rosa and de Pablo, 2011). Brain IR signaling is implicated in satiety, body temperature, reproductive function and aging in several species (Kitamura et al., 2003; Kenyon, 2011; Kleinridders et al., 2014; Ewald et al., 2018). The effect of insulin on neuronal plasticity, through regulation of the uptake, release, and degradation of dopamine and norepinephrine and control of postsynaptic receptor sensitivity (reviewed in Gralle, 2017), has attracted particular attention. There is abundant evidence indicating that disruption of insulin activity in the brain impairs neuronal function and synaptogenesis (Gralle, 2017). Furthermore, roles for IR signaling in glial cell function (including microglia) have been proposed (Kleinridders et al., 2014).

A number of abnormalities found in Alzheimer’s disease (AD) have also been linked to an insulin resistant state, termed “type 3 diabetes” (de la Monte, 2017). Based on the work of multiple research groups conducted in animal models over several decades, an insulin-based therapy for this complex neurodegenerative process has been tested in clinical trials (de la Monte, 2017). Although genetic factors appear to influence the outcome, insulin therapy, particularly when administered intranasally, delayed progression of cognitive impairment in at least a subgroup of AD patients, although side effects in some patients included hypoglycemia. While few retinal studies have been conducted, stimulation of the insulin/mTOR pathway was found to delay cone cell death in a mouse model of retinitis pigmentosa (RP; Punzo et al., 2009). However, in most studies of the effects of insulin on the brain, little attention has been paid to the potential contributions of local proinsulin to the observed outcomes.

Given that expression of the proinsulin gene in the retina of chick embryos leads to production of unprocessed proinsulin (as stated above), and our previous findings demonstrating antiapoptotic effects of proinsulin, via PI3K activation, in mouse retinal cells (Valenciano et al., 2006), in our laboratory we have specifically focused on the potential neuroprotective effects of proinsulin. In a series of preclinical studies conducted using mouse and rat models over the last decade we have provided proof of concept for the use of proinsulin as neuroprotective agent in the treatment of retinal dystrophies, particularly RP (Corrochano et al., 2008; Fernández-Sánchez et al., 2012; Isiegas et al., 2016).

**Figure 2:**
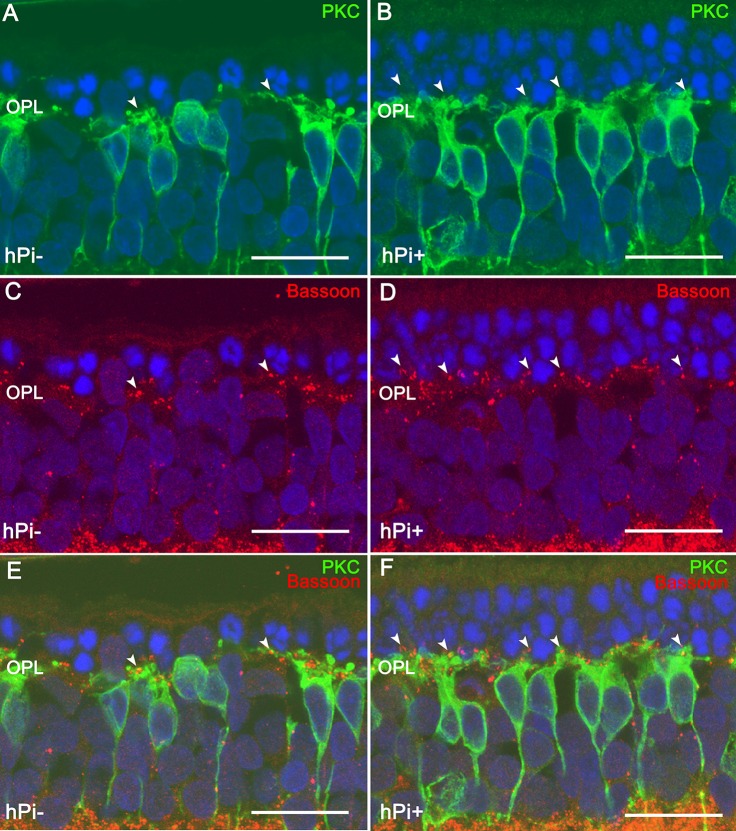
Synaptic connectivity in the retinal outer plexiform layer (OPL) in untreated (hPi−) and proinsulin treated (hPi+) P23H rats. PKC (green) and Bassoon (red) immunostaining reveals preservation of the synaptic connections between the photoreceptors and the bipolar cells in retinas from hPi-treated animals **(B,D,F)** as compared with control rats **(A,C,E)**. Arrowheads indicate cone photoreceptor contacts. Scale bar, 10 μm. Image courtesy of Prof. N. Cuenca reproduced from Fernández-Sánchez et al. (2012).

Inherited retinal dystrophies, including RP, can be caused by a wide variety of mutations in over 300 different genes. In RP in particular, the underlying mutation induces photoreceptor cell death, leading to progressive loss of visual function (Wert et al., 2014). The rd10 mouse, (Chang et al., 2007), is a useful model of autosomal recessive RP in which to assess the potential of proinsulin to attenuate disease progression. We showed that transgenesis of the human proinsulin gene into rd10 mice and its constitutive expression in muscle results in a modest systemic increase in proinsulin levels (low pM range), as well as delayed photoreceptor cell death and attenuated vision loss, as determined by electroretinography (Corrochano et al., 2008). Because this approach is not feasible in a clinical setting, we investigated alternative methods of proinsulin administration. Intraocular treatment of rd10 mice with biodegradable, proinsulin-loaded microbeads proved a successful delivery method, resulting in both cellular and functional neuroprotective effects. Electroretinography revealed increased amplitude of b-cone and mixed b-waves in proinsulin-treated vs. control eyes. Moreover, proinsulin treatment attenuated photoreceptor cell loss, as determined by the thickness and number of cell rows in the outer nuclear cell layer (ONL) of the retina. Proinsulin also increased phosphorylation of AKT^Thr308^ in retinal explants from rd10 mice (Isiegas et al., 2016). In the P23H rat model of autosomal dominant RP, intramuscular injection of an Adeno-Associated Viral (AAV) vector expressing human proinsulin resulted in a significant elevation in levels of circulating proinsulin (with serum levels of 1.1–1.4 nM observed for at least 3 months post-injection; Fernández-Sánchez et al., 2012). The preservation of photoreceptor structure and function confirmed the neuroprotective effect of proinsulin *in vivo*. Moreover, proinsulin treated animals had 49% more photoreceptors than control animals, as well as better preserved synaptic contacts between photoreceptors and bipolar or horizontal cells (Figure 2). Thus, as we await the development of mutation-specific gene therapies, we propose that proinsulin be added to the list of factors that could potentially promote cell survival in retinal dystrophies (Kolomeyer and Zarbin, 2014).

Based on the observation that neurodegenerative diseases of the brain and retina share several underlying pathological changes, including oxidative stress and neuroinflammation (Cuenca et al., 2014; de la Rosa and Hernández-Sánchez, 2019), we investigated the effects of injection of AAV vector bearing the human proinsulin gene in the SAMP8 mouse, a model of precocious brain senescence that exhibits several Alzheimer’s disease-like traits (Corpas et al., 2017). Proinsulin treatment activated the AKT pathway and decreased expression of neuroinflammation markers in the hippocampus 5 months after injection. Moreover, proinsulin-treated mice exhibited lower levels than untreated controls of TNF-α, interleukin-1β and the antiprotease α2-macroglobulin in the hippocampus, as well as decreased GFAP immunoreactivity, which reduced astrocyte reactivity to control levels. Importantly, these neuroprotective effects correlated with improved cognitive performance in spatial and recognition tasks. Interestingly, in these mice the concentration of circulating proinsulin after a single injection of the AAV vector was rather low (13 pM), and glycemia was unchanged with respect to untreated animals. Moreover, the absence of detectable human insulin in the treated mice confirmed that conversion of proinsulin to insulin was minimal. It will be interesting to further study neuroinflammatory mechanisms involving microglial cells in physiological and pathological aging as well as in retinal dystrophy models, and the response to proinsulin treatment.

The observations recapitulated above support our view that proinsulin is a suitable neuroprotective therapy, with lower metabolic risks than insulin; in our studies there were no changes in mice or rats body weight or glycemia. In the only clinical trial conducted to date (Galloway et al., 1992), proinsulin was tested as a possible insulin agonist of intermediate-acting effect for the treatment of diabetes mellitus. Very high levels of serum proinsulin were reached (1.4–8 nM, almost a 1,000-fold increase with respect to normal values), raising concerns of an increased risk of myocardial infarction which led to the disclaimer of the future use of proinsulin in that metabolic context. Interestingly, however, after 6 months of extreme hyperproinsulinemia, no progress of diabetic retinopathy was observed. In our own studies we showed that proinsulin exerts neuroprotective activity at much lower concentrations, starting at the low pM range (Corrochano et al., 2008; Corpas et al., 2017). Moreover, because neurons are postmitotic cells and proinsulin does not bind to the IGF-1 receptor, the likelihood of unwanted proliferative side effects is significantly reduced. In summary, proinsulin may constitute a safer alternative to insulin for activation of the IR-A pathway in the context of neuronal degeneration or aging, especially if administered directly to the retina or brain. Further clinical trials will be required to determine the appropriate dose for each condition and the optimal route of administration.

## Future Prospects

To translate the experimental observations to the clinical practice, preclinical studies with proinsulin using larger and preferentially diurnal animal models of RP, possibly using local administration of AAV vector in the eye, will need to be conducted before clinical trials are feasible. The advantages of proinsulin vs. insulin (longer half-life, attenuated AKT activation, very low risk of hypoglycemic or proliferative secondary effects), and the availability of several delivery methods to reach the target cells, suggest a promising future for proinsulin as a neuroprotective factor in retinal neurodegenerative diseases. Two major drawbacks are the current lack of a commercial proinsulin preparation suitable for immediate testing and the requirement of GMP-level formulations for advanced preclinical studies. Lessons learned in studies of rare diseases such as RP may be useful in other contexts in which neuroprotection could be beneficial (e.g., the use of proinsulin in combination with other therapies, including mutation-specific gene therapy), paving the way for broader use of proinsulin in the treatment of a range of more common conditions such as in glaucoma or the inflammaging brain.

## Author Contributions

FP wrote the article. CH-S and ER contributed to the final revision and editing of the text and figures.

## Conflict of Interest Statement

The authors declare that part of the research was performed after they were founders and scientific advisors of the CIB-CSIC spin-off company ProRetina Therapeutics S.L. presently extinguished.
